# Cost-effectiveness of early intervention in psychosis in Latin America: economic evaluation of Chilean services

**DOI:** 10.1192/bjo.2026.11033

**Published:** 2026-05-09

**Authors:** David Aceituno, Nicolas A. Crossley, Huajie Jin, Carlos Balmaceda, Eduardo A. Undurraga, Alfonso Gonzalez-Valderrama, Paul McCrone, Matthew Prina, Mark W. Pennington, for the ANDES Network

**Affiliations:** Health Service and Population Research, King’s College London, London, UK; Department of Psychiatry, Pontificia Universidad Catolica de Chile, Santiago, Chile; School of Government, Pontificia Universidad Catolica de Chile, Santiago, Chile; Surveillance, Epidemiology, and New Technologies for Infectious Emerging Threats (SENTINET), Santiago, Chile; School of Medicine, Universidad Finis Terrae, Santiago, Chile; Institute for Lifecourse Development, University of Greenwich, London, UK; Faculty of Medical Sciences, Newcastle University, Newcastle, UK; Source Health Economics, London, UK; Epsilon Research, Santiago, Chile; Centre for Research on Health and Social Care Management (CERGAS), Bocconi University, Milan, Italy

**Keywords:** Early intervention in psychosis, low- and middle-income countries, Latin America, economic evaluation, cost-effectiveness analysis

## Abstract

**Background:**

International evidence suggests that Early Intervention for Psychosis (EIP) services are both effective and cost-effective. Such evidence, however, comes almost exclusively from high-income countries.

**Aims:**

Our aim was to estimate the cost-effectiveness of EIP services in a Latin American setting.

**Method:**

We compared EIP services against community mental health teams (CMHT) from the Chilean health system perspective. We developed a six-state Markov model to estimate the costs, benefits (measured as quality-adjusted life-years (QALYs)) and incremental cost-effectiveness ratio (ICER) for a 10-year time horizon. The model was populated with data from a Chilean EIP cohort, published literature and expert opinion. We characterised uncertainty through probabilistic sensitivity analysis and calculated the value of information to reduce such uncertainty.

**Results:**

In the base case analysis, EIP was cost-effective compared with CMHT, with an ICER of 5 550 044 Chilean pesos per QALY (USD 13 742 adjusted for purchasing power parity). Uncertainty analysis revealed an 80% probability of EIP services being the most cost-effective option at a willingness-to-pay threshold of one gross domestic product per capita (USD 15 923). Sensitivity analysis showed that the results were sensitive to parameters such as intervention effectiveness and cost, suggesting that a new trial might be worthwhile to reduce uncertainty.

**Conclusions:**

This model suggests that implementing EIP services in Chile may cost more, but it is likely to be cost-effective. Nonetheless, more evidence about affordability, equity and broader perspectives is needed to improve the economic case of implementing EIP services in less-resourced settings, such as in Latin America.

The favourable evidence of effectiveness and cost-effectiveness of Early Intervention for Psychosis (EIP) services has motivated their implementation worldwide.^1^ Such evidence, however, comes almost entirely from high-income countries (HICs).^2–4^ In low- and middle-income countries (LMICs) the duration of untreated psychosis (DUP) is longer and the treatment gap higher.^5^ Therefore, it is crucial to generate local evidence to adequately implement this model of care.^6^

An adequate implementation should align service availability with people’s needs in a timely manner. Such services should be sensitive to the context, promoting recovery through engaging, collaborative rapport and continuity of care. Additionally, these services might require adaptation to the local culture, but keeping interventions as close as possible to the original protocols to secure fidelity.^7^ Finally, a sustainable scaling-up requires sufficient and continuous funding.^8^

Because resources are limited, implementing a new intervention imposes an opportunity cost on those who do not receive care, on account of resources being invested elsewhere.^9^ In this case, if EIP services are not cost-effective in the health systems where they are being implemented, such resources can be better used elsewhere. Furthermore, once services are established, disinvestment can be difficult and poorly managed changes may disrupt care pathways. On the other hand, not implementing interventions that are cost-effective is an inefficient use of resources and hinders people from receiving useful interventions.^9^

Chile is a Latin American country with approximately 20.1 million inhabitants. Although considered a HIC since 2013 by the World Bank, the country presents high levels of inequality and poverty. Mental disorders are responsible for nearly 10% of the burden of diseases,^10^ and a recent study estimated the incidence of first-episode of psychosis at 18.9 (CI: 18.7–19.1) per 100 000 person-years.^11^ The treatment of schizophrenia is prioritised by an explicit health benefit plan known as *Garantias Explícitas en Salud* (GES). This includes pharmacological and psychosocial interventions provided by community mental health teams (CMHT) at secondary care. Although such a package of care is backed up by evidence of cost-effectiveness,^12^ in practice, patients attending CMHT receive medications but rarely other types of intervention.^13^

This situation has motivated the development of services specially dedicated to help people in the early stages of psychosis.^14^ Such developments are similar to pioneering EIP initiatives in Australia and England, where these services are now provided nationwide. However, before expanding these services to the national level in Chile, local evidence is paramount. The importance of local evidence in LMICs has been highlighted by a recent review that found EIP interventions in only three LMICs (India, Iran and Uganda).^4^ The authors observed that no information about the cost-effectiveness of EIP services in LMICs could be found. Since that review was conducted, an economic evaluation of Brazilian EIP services has been published^15^ which, to the extent of our knowledge, is the only cost-effectiveness analysis of an EIP service in a LMIC.

Given the scarcity of economic evidence of EIP services derived from LMICs, despite the recognised importance of local evidence, our aim was to estimate the cost-effectiveness of implementing EIP services within the Chilean health system.

## Method

### Study design

We carried out an economic evaluation comparing EIP services against CMHT from the health system perspective following current methodological guidelines.^16^ This economic evaluation was a cost-utility analysis using a decision analytical model, following the guidance in the ISPOR-SMDM Task Force reports.^17^ This report also complies with the updated Consolidated Health Economic Evaluation Reporting Standards (CHEERS) guidelines for economic evaluations.^18^

### Study population

The population of interest was people aged 15–35 years with a first episode of psychosis (FEP). This includes the category of schizophrenia and other primary psychotic disorders according to ICD-10. Psychoses due to affective disorders (bipolar disorder and depression) are covered by other guidelines, and were excluded from this evaluation. Psychoses secondary to medical conditions or substance abuse were also excluded.

### Interventions

The intervention consisted of an EIP service according to international consensus statements, implemented in an urban area of Santiago.^19^ These services comprise a multidisciplinary team providing mental healthcare exclusively for people with a FEP, with a case-to-client ratio lower than that of standard care. The specific interventions include low-dose antipsychotics, psychological therapy, family psychoeducation and vocational support. Furthermore, the clinical teams implement internal audit and regular meetings as recommended by EIP fidelity scales.^7^ The comparator was treatment by CMHT. These services provide interventions for people with schizophrenia according to national guidelines, but they are not exclusively aimed at the early stages of psychosis and professionals must deliver care for people with other mental disorders.

### Economic outcomes

Economic outcomes included the following: (a) costs measured in 2018 Chilean pesos (CLP); (b) benefits measured as quality-adjusted life-years (QALYs); and (c) incremental cost-effectiveness ratio (ICER) according to the following equation:
(1)

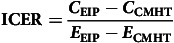




where *C*_EIP_ is mean costs of EIP, *C*_CMHT_ is mean costs of CMHT, *E*_EIP_ is mean effectiveness of EIP and *E*_CMT_ is mean effectiveness of CMHT

Additionally, we used the net benefit approach to account for uncertainty in ICERs. The net benefit approach includes a theoretical (although unknown) value (*λ*) that society would place on one unit in improvement in the outcome of interest.^20^ This approach overcomes the limitations in the interpretation of ICER confidence intervals, and allows comparisons on the same scale:
(2)

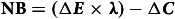




where NB is the net benefit approach, *Δ* ***E*** is differences in effects, *Δ* ***C*** is differences in costs and ***λ*** is willingness to pay.

At the moment, because Chile does not have an explicit cost-effectiveness threshold to specify ***λ***, as a benchmark we utilised the World Health Organization (WHO) recommendation of using 1–3 times gross domestic product (GDP) per capita (USD 15 923). However, because such a threshold has been criticised for overestimating the cost-effectiveness of interventions, we tested lower values of ***λ*** in the sensitivity analysis.

A 10-year time horizon was considered appropriated in the base case analysis, because it is the longest follow-up from a randomised controlled trial registered in the literature.^21^ Longer time horizons, including 20 years, 30 years and lifetime horizon (considering an average life expectancy of 80 years in the Chilean population), were evaluated in the sensitivity analysis. Costs and effects were discounted at 3.0% to reflect time preference by valuing future costs and health outcomes slightly less than present ones, in line with standard methodological recommendations.^16^

### Economic model

#### Model conceptualisation

**Fig. 1:**
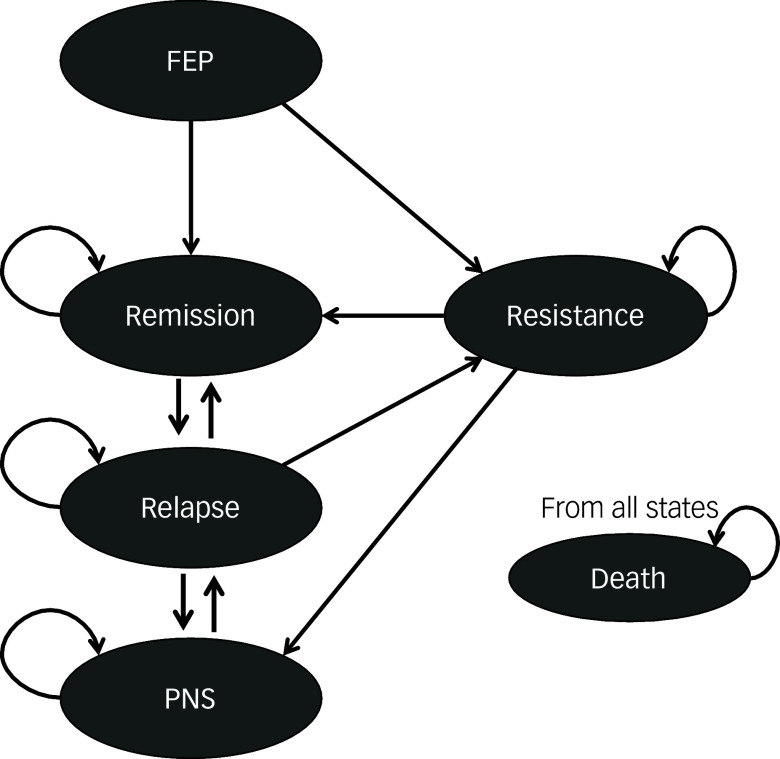
Markov model conceptualisation. FEP, first-episode psychosis; PNS, persistent negative symptoms.

Model selection followed an iterative approach, including inputs from local stakeholders (clinicians and policy-makers), Chilean clinical guidelines and a systematic review of decision models covering the entire pathway of care for people with schizophrenia.^22^ The model comprised a transition model of six health states, also known as a Markov model, with 3-month cycles capturing states considered relevant to the decision context. Briefly, a Markov model is a mathematical model using transition probabilities that represents patients moving between mutually exclusive health states over time, to estimate long-term costs and health outcomes.^17^ A schematic representation can be found in Fig. 1.

According to this model, a cohort of FEP patients moves between mutually exclusive health states defined by transition probabilities. People with a FEP experience variable degrees of symptom remission, which was defined by the Positive and Negative Syndrome Scale (PANSS) according to the Andreasen criteria.^23^ Those who do not recover can develop treatment-resistant schizophrenia if they do not respond to two trials of antipsychotic medications at 600 mg equivalent of chlorpromazine, as recommended by consensus guidelines.^24^ People achieving remission can either remain in such a state or suffer a symptomatic relapse, defined as worsening of symptoms requiring clinical care;^25^ a proportion of these patients will require inpatient care to achieve symptom stability. Additionally, we added a health state to include patients with persistent negative symptoms; these have been recognised as an independent source of disability despite control of positive symptoms.^26^ Finally, according to the model, people can die from every health state with a probability equal to their age-specific mortality rate (derived from Chilean life tables) multiplied by the hazard ratio associated with each health state.

As in any model, we excluded health states described in the natural history of the disease but deemed less relevant to the decision setting. For instance, the utility of services for people at clinical risk of psychosis remains controversial^27^ and incipient only in Chile. A detailed list of assumptions and simplifications can be found in the Supplementary material.

#### Model parameters and data sources

The parameters used to populate the model were estimated from a Chilean cohort of FEP patients, published literature, local cost data and expert opinion.

The transition probabilities in the baseline natural history model (CMHT arm) were obtained by conducting a rapid review for each health state. Rapid reviews are structured evidence syntheses that intentionally abbreviate certain steps (e.g. narrower searches, single screening, prioritised study designs) while retaining prespecified methods and transparent reporting, consistent with Cochrane Rapid Reviews Methods Group guidance.^28^ When selecting a study to feed the model we followed published recommendations, favouring systematic reviews, then primary observational studies and then the control arm of randomised trials.^29^ When no evidence for a certain parameter could be found, we elicited it from experts through a Delphi panel, as detailed in the supplementary materials.

The transition probabilities in the intervention arm were estimated from a cohort study taking place in the EIP service at the Psychiatric Institute Dr Jose Horwitz Barak in Santiago, Chile. Patients who agreed to participate were interviewed by research assistants certified in the standardised assessment of psychosis. Data collection took place between 2017 and 2019, and included assessments at baseline and 3-month follow-up. The data items included sociodemographic characteristics, anthropometric measures, ICD diagnosis, medications, substance abuse, PANSS scores, Hamilton Rating Scale for Depression, Young Mania Rating Scale and a cognitive evaluation using the MATRICS consensus battery.^30^

Because of the observational nature of the sample, we pooled individual patient data with those from previously published meta-analyses using a Bayesian synthesis approach known as power prior.^31^ This method has been used when combining different sources of evidence to account for the higher risk of bias of observational evidence, while preserving its information relevant to the decision setting.^31^ We applied this method in a random-effects binomial model to account for the nested nature of the data. We assumed exchangeability of the studies, and the results were embedded in the Markov model using the log risk ratio scale. Details of the Bayesian analysis can be found in the Supplementary material.

In order to calculate the benefits associated with the interventions, each health state is associated with a specific health-related quality of life utility value (HSUV). HSUVs were extracted after conducting a systematic review.^32^ The total number of QALYs gained by each strategy was obtained by multiplying non-dead health states by their respective HSUVs at each cycle and then summing these across the entire time horizon. The discount rate was applied to cycle length following previously suggested adjustment.^9^

Finally, the cost parameters associated with each health state were estimated by multiplying local unit costs by their resource use. Chilean unit costs are published every 2 years by the Ministry of Health, and include all interventions and actions related to health problems included in the GES plan.^33^ This includes hospitalisation costs, visits to emergency departments and appointments with professionals at different levels of care. Medication costs were obtained from the national supply centre for drugs (CENABAST). Given the absence of patient-level data in terms of resource use, identification and measurement of resource items was derived from published systematic reviews^34^ and validated by local stakeholders (see Supplementary material). All costs are reported in 2018 Chilean currency (CLP). To facilitate international comparisons, we converted CLP to US dollars and UK pound sterling (GBP), adjusted for purchasing power parity (PPP) conversion rates provided by the Organisation for Economic Co-operation and Development.

A list of the model’s parameters, including point estimates, probability distributions and sources, is provided in the Supplementary material.

#### Sensitivity analysis

We conducted a series of deterministic and probabilistic sensitivity analyses (PSA) to assess the robustness of the model results. In PSA we ran the model for 5000 iterations using Monte Carlo simulation to account for uncertainty in the parameters.

Additionally, we conducted value of information (VOI) analysis to assess the potential value of additional research.^35^ Briefly, given the uncertainty associated with the input parameters, there is always a possibility that the decision made with the current information is suboptimal. In theory, this uncertainty can be reduced by gathering more information. However, this process is also costly and uncertain. To deal with this situation, VOI analysis provides a formal assessment of the value of additional research, based on the extent to which the decision is improved by reducing uncertainty compared with the cost of new research. Following current methodological recommendations,^35^ we calculated the expected value of perfect information (EVPI) for the population affected by the intervention using a recent estimation of the incidence of FEP in Chile.^11^ Additionally, we calculated partial EVPI to assess which parameters were more influential on the decision.

#### Model implementation

The model was fully implemented in the programming language R, version 4.2.1 (2022) for MacOS (R Foundation for Statistical Computing, Vienna, Austria; https://cran.r-project.org/bin/macosx/), following coding good-practice recommendations.^36^ Bayesian analysis was implemented using the software Just Another Gibbs Sampling. We applied white- and black-box tests to verify and validate the internal results of the model.^17^ Face validity was provided by local stakeholders. Details of the verification tests are reported in the Supplementary material, and detailed code is available at https://github.com/david-aceituno.

### Ethical standards

The primary study used to populate this model was conducted according to international ethical regulations, and was approved by the ethics committee of Pontificia Universidad Católica de Chile (approval no. 190603005). All adult participants (aged >18 years) signed an informed consent. For younger participants, patient and legal tutors’ consent was required.

## Results

### Study parameters

Individual-level data from the Chilean cohort study comprised 82 patients with FEP; most (78.5%) were male, with a mean (s.d.) age of 20.2 (3.49) years and 12.3 years of education. At baseline, the mean (s.d.) PANSS score was 33.26 (9.35) and the depression mean (s.d.) score measured by the Hamilton Rating Scale for Depression was 6.86 (4.76). Characteristics of the study participants can be found in Table 1.

**Table 1:** Baseline characteristics of participants

Characteristic	*n*	%
Gender		
Male	62	75.6
Female	20	24.4
Age, years (mean, s.d.)	20.3	3.5
Education, years (mean, s.d.)	12.3	2.3
Marital status		
Single	82	100
Married	0	0
Widowed	0	0
Civil partner	0	0
Missing	11	13.4
Clinical		
PANSS positive (mean, s.d.)	16.9	6.3
PANSS negative (mean, s.d.)	20.3	8.8
PANSS total (mean, s.d.)	33.3	9.4
HDRS (mean, s.d.)	6.9	4.8
YMRS (mean, s.d.)	5.6	4.0
BMI (mean, s.d.)	22.9	3.8
Missing	5.0	6.1

PANSS, Positive and Negative Symptoms Scale; HDRS, Hamilton Depression Rating Scale; YMRS, Young Mania Rating Scale; BMI, body mass index.

**Fig. 2:**
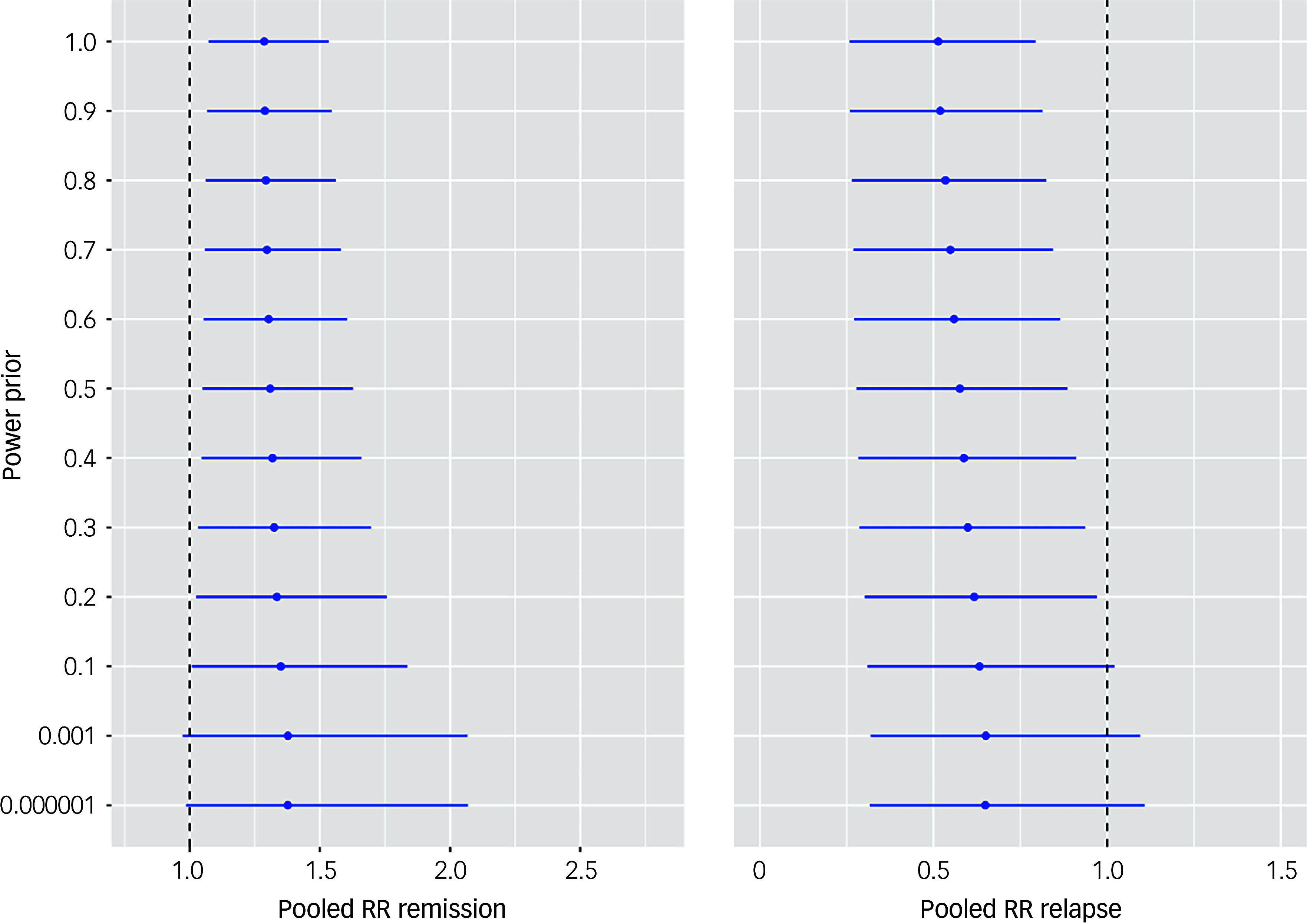
Pooled risk ratios (RRs) at different levels of inclusion of observational evidence. Forest plots showing the effect of incorporating observational evidence within a meta-analysis of aggregate data. The *y* axis shows increasing weighting of the observational evidence from bottom to top, because the weighting factor is exponential. The *x* axis represents the estimated effect size (remission at left and relapse at right) in the RR scale. The blue point-and-range lines represent pooled effect sizes at different weightings of the observational evidence.

The aggregate data included 7 studies with 2087 participants. Six studies evaluated the effectiveness of EIP services in achieving remission, and six assessed relapse prevention (five studies reported both outcomes). The effect of including cohort data is presented in Fig. 2. The results of the Bayesian model showed that people receiving EIP had a pooled risk ratio of 1.25 (95% credible interval (CrI): 1.05–1.58) of achieving remission when the observational evidence was fully incorporated. When the observational evidence was downweighted by 50%, the risk ratio changed slightly to 1.27 (95% CrI: 1.02–1.58); meanwhile, risk ratio was 1.33 (95% CrI: 0.93–2.06) when the observational data were discarded (power prior of 0). When the outcome assessed was relapse, the pooled risk ratio in the EIP group was 0.51 (95% CrI: 0.25–0.79) when the observational evidence was considered an additional trial. The risk ratio increased to 0.57 (95% CrI 0.28–0.88) when the individual-participant data were downweighted by 50%, and to 0.70 (95% CrI 0.36–1.13) when this type of evidence was excluded. Full details of the Bayesian synthesis, including model convergence diagnostics and forest plots, can be found in the Supplementary material.

### Base case analysis

The results of the base case analysis are shown in Table 2. According to this analysis, the implementation of EIP services resulted in a mean incremental cost of CLP 1 327 054 and a mean incremental benefit of 0.24 QALYs. The resulting ICER of CLP 5 550 044 (USD 13 742 or £9465 adjusted for PPP) per QALY was considered cost-effective at 1 GDP per capita of willingness-to-pay (WTP) (USD 15 923 or £10 968 PPP adjusted).

**Table 2:** Results of base case analysis comparing early intervention for psychosis services against community mental health teams

Strategy	Costs (CLP)	Effects (QALYs)	Increased costs (CLP)	Increased effects (QALYs)	ICER
CMHT	15 652 985	5.95	NA	NA	NA
EIP	16 980 039	6.18	1 327 054	0.24	5 550 044

CLP, Chilean peso; QALYs, quality-adjusted life-years; ICER, incremental cost-effectiveness ratio; CMHT, community mental health teams; EIP, Early Intervention for Psychosis; NA, not applicable.

### Sensitivity analysis

**Fig. 3:**
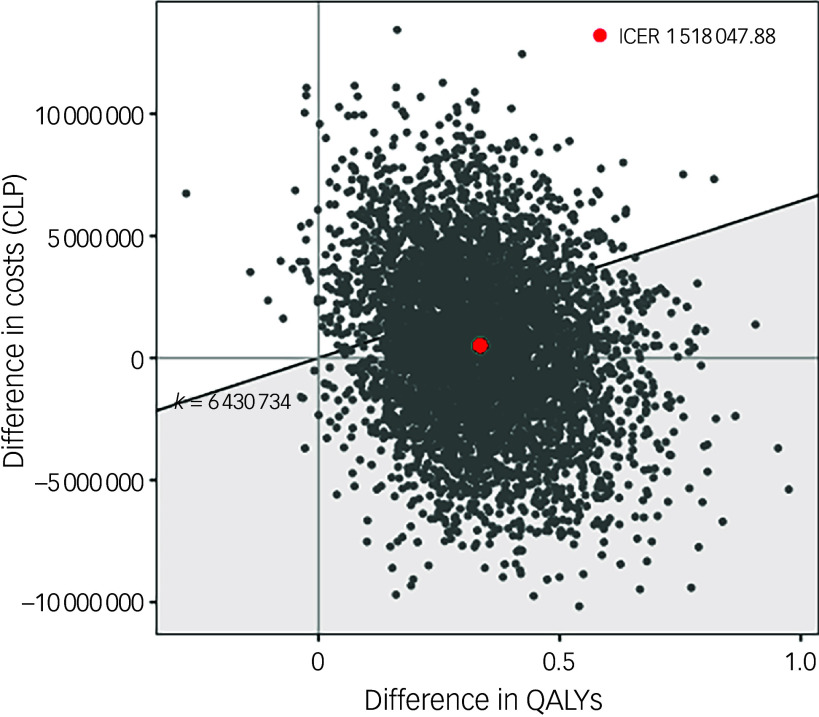
Cost-effectiveness plan showing PSA simulations. The *x* axis represents the difference between EIP services and CMHT in terms of QALYs; the *y* axis represents the difference between EIP services and CMHT in terms of costs (CLP). PSA, probabilistic sensitivity analysis; EIP, Early Intervention in Psychosis; CMHT, community mental health teams; QALYs, quality-adjusted life-years; CLP, Chilean peso; ICER, incremental cost-effectiveness ratio.

The results of the 5000 simulations in PSA are plotted on the cost-effectiveness plane in Fig. 3. PSA showed that most (82.5%) of the simulation lay in the north-east quadrant followed by 9.6% in the south-east quadrant, where EIP dominates; 7.3% of the simulation lay in the north-west quadrant, where CMHT dominate.

Deterministic sensitivity analyses revealed that the model was sensitive to the following parameters: HSUVs of remission, HSUVs of resistance, probability of remission following an acute episode and probability of relapse given remission. However, EIP remained as the most cost-effective alternative compared with CMHT. Full details of the sensitivity analysis are presented in the Supplementary material.

**Fig. 4:**
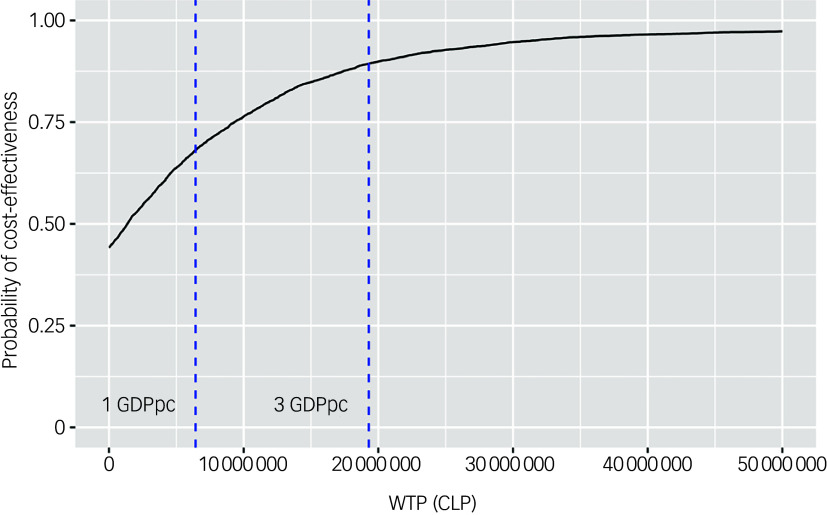
Cost-effectiveness acceptability curve showing the probability of EIP being cost-effective at different thresholds of WTP. The *x* axis shows WTP in Chilean peso (CLP), with the *y* axis representing the probability of cost-effectiveness. EIP, Early Intervention in Psychosis; WTP, willingness to pay; GDPpc, gross domestic product per capita.

Using the net benefit approach, EIP was associated with the highest expected net benefit at all WTP thresholds. In the base case analysis, at 1 GDP per capita (15 923 USD) of WTP, EIP had a probability of 0.85 of being the alternative with the highest expected net benefit. When the price that society is willing to pay for one increment in QALYs in people with FEP was halved, the probability of EIP being the most cost-effective alternative was 0.80. The cost-effectiveness acceptability curve shows the probability of EIP being cost-effective compared with CMHT at different thresholds of WTP (Fig. 4).

### VOI

The VOI analysis revealed that, in the base case analysis, the uncertainty surrounding cost-effectiveness translates into CLP 805 857 (£1374) of decision uncertainty. When the total population affected by the decision is taken into account (which included the annual incidence of FEP over a 10-year time horizon), the population EVPI reached CLP 26.1 billion (£44 532 358), indicating that further research might be valuable if costs are less than such an amount. Additionally, partial EVPI suggested that the effectiveness estimates and utility values of people in remission have the highest influence on this estimate. Furthermore, the expected value of partial perfect information analysis suggested that the parameters causing most of the decision uncertainty were the effectiveness of the intervention in reducing relapses, the costs of managing those relapses, the effectiveness of the intervention promoting remission and the costs associated with remission state. Further details of VOI analyses can be found in the Supplementary material.

## Discussion

In this work we have evaluated the potential costs and benefits of implementing specialist services for people with FEP in the Chilean healthcare system. The results of this modelling exercise suggest that EIP services are a more efficient use of resources compared with standard care. Applying the commonly used WHO threshold of 1–3 GDP per capita (15 923–47 769 USD), EIP services had a chance of >80% of being more cost-effective than CMHT, according to the incremental analyses and net benefit approach.

### Comparison with published literature

Comparisons between economic evaluations from different jurisdictions can be problematic due to differences in health system organisation, economic perspectives and study designs. Despite these differences, the results of our model are in alignment with published evidence of the cost-effectiveness of EIP.^3,37^ Several trial- and model-based economic evaluations have shown that implementation of EIP services is cost-effective.

However, as we previously highlighted, most of the evidence comes from HICs, with pioneering work in Australia and further evidence from Europe, North America and Asia.^3^ Given that resources are even more limited in LMICs, having good cost-effectiveness estimates is crucial. However, producing local evidence in LMICs is difficult because of low investment in research, scarcity of reliable data and the cost of research itself.^8^ In these situations, decision models can be used to support rational decision-making by simulating the potential benefits of alternative interventions.^17^ Furthermore, models allow quantification of the uncertainty of the decision and measure the value of additional research. In mental health, several recommendations have been made on the basis of modelling exercises (e.g. WHO-CHOICE research).^38^ Our group recently presented a model-based economic evaluation of EIP services in Brazil, with results similar to those for the Chilean case presented here.

### Strengths and limitations

This study has both strengths and limitations. First, our model development followed a systematic and iterative approach to include the best published evidence, combined with Chilean data and opinions from local experts. We also conducted a wide range of sensitivity analyses, propagated uncertainty throughout the model, tested several scenarios and estimated the value of conducting further research. Second, we were able to use local cost data, which are updated regularly based on the schizophrenia GES benefit plan. Third, using a Bayesian approach allowed the inclusion of local cohort data in a rational and transparent manner. Cohort data have the potential of improving the applicability of findings, whereas their higher risk of bias can be downweighted using the power prior approach.

However, several limitations need to be acknowledged. First, the cohort sample size was small and with a short period of follow-up. This might partially explain the small impact of the observational data on the overall evidence. Second, the sample did not include a measure of DUP. It is, therefore, unclear whether such a variable might have changed the effectiveness of EIP services in Latin America, as suggested elsewhere.^39^ Third, our model was limited to participants with non-affective psychosis. In practice, people with bipolar disorder and/or depression with psychotic features might present to EIP services, but in the Chilean context they are covered by a separate GES package of care.

Fourth, although unit costs were available for most of the interventions included in EIP services, no patient-level data about service use could be found. As a result, we relied on administrative data and external literature to estimate service use. Despite using nationally validated unit costs, the measurement of service use in Chile is an area that requires further research.

Fifth, given the characteristics of the Chilean health system, it was not possible to measure private sector charges and therefore these results can be applied only to the public health sector. Although the public sector represents the majority of the healthcare system, at least 15% of the Chilean population have the private sector as their main provider of care, where price data are inaccessible to researchers and civil society.

Sixth, the model development includes only expert knowledge from clinicians and researchers. We did not include the inputs of experts by experience and/or their caregivers, which might have had relevant implications by changing the health states that matter most to those affected by psychosis.

Finally, restricting the perspective to the healthcare system has the limitation of underestimating the benefits of interventions with consequences outside the health system. This is especially relevant in mental health, where productivity loss, social care expenses and costs to caregivers are particularly relevant.^3^

### Policy implications and further research

The results of this cost-effectiveness analysis suggest that EIP services are cost-effective compared with CMHT. At the health policy level, these results have direct implications for the Chilean GES plan. For instance, the package of care currently provided by the GES plan would need to be expanded to closely align with those provided by EIP teams. Furthermore, our results suggest that treatments for people with FEP might be better implemented as a stand-alone service rather than within CMHT. Nevertheless, such implementation would require further examination of potential unintended consequences, such as the effect of including specialist services in the remainder of the mental health network, potential understaffing of CMHT and diversion of resources. A careful implementation is crucial to preserve the economic benefits of this intervention.^1,37^

Additionally, the VOI analysis suggested that there is potential value in reducing uncertainty by collecting more data, particularly for certain parameters such as those related to the effectiveness of EIP programmes and the costs of remission. In addition to reducing this uncertainty regarding parameters, there is value in conducting further research prior to a wider implementation, despite evidence of cost-effectiveness. A recognised problem when moving from research to policy decisions is affordability.^8^ Many LMICs face the problem of having good evidence of cost-effectiveness but with no budgets to set up new programmes or interventions. In such cases, a budget impact analysis would be useful to add information on the costs of implementation.

Similarly, no information about the impact of EIP services on health inequalities has been produced. There is consistent evidence of huge health disparities among people with mental health problems, especially psychosis.^40^ However, to date no published study has examined the potential impact of implementing EIP services on such disparities.

Overall, our results suggest that implementing EIP services in Chile could be cost-effective, although further research incorporating patients’ point of view, measures of health inequalities and a societal perspective is warranted to ensure a sustainable scaling-up.

## Supporting information

10.1192/bjo.2026.11033.sm001 Aceituno et al. supplementary material Aceituno et al. supplementary material

## Data Availability

The authors declare that all data supporting the findings of this study are available within the article, its supplementary information file and the GitHub repository.

## Supplementary material

The supplementary material is available online at https://doi.org/10.1192/bjo.2026.11033
