# Supplementary material for: Cost-effectiveness of early intervention in psychosis in Latin America: economic evaluation of Chilean services
Source: BJPsych Open. 2026 May 9;12(3):e124. doi: 10.1192/bjo.2026.11033 (PMC13150720; doi:10.1192/bjo.2026.11033)
Supplement: Aceituno et al. supplementary material [file S2056472426110333sup001.docx]

Supplementary materials

Early intervention in psychosis in Latin America: cost-effectiveness analysis of Chilean services

Table of Contents

[Model conceptualisation 3](#_Toc220571826)

[Model parameters 5](#_Toc220571827)

[Bayesian synthesis 8](#_Toc220571828)

[Expert elicitation 9](#_Toc220571829)

[Justification 9](#_Toc220571830)

[Methods 10](#_Toc220571831)

[Experts selection 10](#_Toc220571832)

[Evidence dossier 10](#_Toc220571833)

[Elicitation exercise 10](#_Toc220571834)

[Statistical analysis 11](#_Toc220571835)

[Ethical issues 11](#_Toc220571836)

[Results 12](#_Toc220571837)

[The exercise 12](#_Toc220571838)

[Data aggregation 13](#_Toc220571839)

[Usage of elicited quantities in the model 16](#_Toc220571840)

[List of black-box and white-box tests 16](#_Toc220571841)

[Deterministic sensitivity analysis 17](#_Toc220571842)

[Value of information analysis 18](#_Toc220571843)

[Other scenarios 20](#_Toc220571844)

[References 22](#_Toc220571845)

# Model conceptualisation

Figure S1 shows a comprehensive diagram of the natural course of psychotic disorders based on the published literature ^1–3^ and clinical inputs. The arrows signal the patients’ pathways. Some patients can stay in the same health state at the end of a cycle, meanwhile other can move to other states based on transition probabilities. The figure also shows states and events excluded from the model (in transparent) meanwhile health states modelled are in bolded font. Below, are presented simplifications and assumptions.

Figure S1 Detailed model conceptualisation

Model simplifications:

- Exclusion of clinical high-risk of psychosis (CHR-P).
- We did not include the adherence to the interventions.
- We did not cost expenses and consequences outside the healthcare sector.
- Some researchers have highlighted the distinction between symptomatic remission and recovery ^2^. The latter would be a “personal process of overcoming the negative impact of diagnosed mental illness/distress despite its continued presence”. We did not explicitly modelled recovery.
- Mortality was estimated from Chilean life tables times the standardised mortality ratio (SMR) estimated from a recent meta-analysis ^4^. This include mortality attributable to natural and unnatural causes.

Model assumptions:

- We assumed comorbidities were similar in both groups. We found no evidence of EIP attending less or more severe patients.
- Patients receive comparable pharmacological interventions. There are some evidence people attending EIP services receive less polypharmacy which is associated to more adverse effects ^5^. Therefore, this assumption might underestimate potential harms in the comparator.
- Relapse is equivalent to the first-episode psychosis in terms of symptomatology.
- After remission, patients need to have a relapse in order to develop treatment-resistant schizophrenia (TRS) or persistent negative symptoms (PNS).
- Resistance is assumed similar to relapse in terms of positive symptoms, therefore the transition to relapse is zero.
- Health utilities for remission is equivalent to the general population of similar age.
- Health utilities for relapse is equivalent to a severe state according to this systematic review.
- Health utilities for PNS is equivalent to a moderate state according to this systematic review.
- Standardised mortality rate is the same in both arms. Although there is incipient evidence of lower risk of suicide in people receiving EIP services, we considered this evidence as insufficient to include it in the model.

# Model parameters

Table S1 List of input parameters

| Parameter | Mean value | Low value | High value | Distribution | Source |
| --- | --- | --- | --- | --- | --- |
| **Transition probabilities** | | | | | |
| Probability of remission after FEP | 0.167 | 0.054 | 0.237 | beta(𝛼 = 29.72, 𝛽 = 147.57) | ^6^ |
| Probability of remission after FEP (calibrated) | 0.182 | 0.161 | 0.198 | beta(𝛼 = 39.13, 𝛽 = 175.67) | Calibration |
| Probability of resistance after FEP | 0.059 | 0.041 | 0.073 | beta(𝛼 = 4.93, 𝛽 = 78.68) | ^7^ |
| Probability of PNS after FEP | 0.107 | 0.077 | 0.140 | beta(𝛼 = 25.59, 𝛽 = 212.91) | Experts elicitation^1^ |
| Probability of relapse after remission | 0.078 | 0.062 | 0.093 | beta(𝛼 = 62.41, 𝛽 = 735.66) | ^8^ |
| Probability of remission after resistance | 0.132 | 0.076 | 0.208 | beta(𝛼 = 9.34, 𝛽 = 61.02) | ^9^ |
| Probability of PNS after resistance | 0.107 | 0.077 | 0.140 | beta(𝛼 = 25.59, 𝛽 = 212.91) | Assumption^2^ |
| Probability of relapse after PNS | 0.078 | 0.062 | 0.093 | beta(𝛼 = 62.41, 𝛽 = 735.66) | ^8^ |
| Probability of remission after PNS | 0.147 | 0.103 | 0.196 | beta(𝛼 = 22.50, 𝛽 = 130.80) | ^10^ |
| Probability of receiving Clozapine if TRS in CMHTs | 0.163 | 0.119 | 0.211 | beta(𝛼 = 27.71, 𝛽 = 142.29) | ^11^ |
| Probability of receiving Clozapine if TRS in EIP | 0.620 | 0.246 | 0.942 | beta(𝛼 = 2.37, 𝛽 = 1.45) | Experts elicitation |
| Probability of hospitalisation if relapse | 0.357 | 0.109 | 0.661 | beta(𝛼 = 2.50, 𝛽 = 4.45) | Experts elicitation |
| **Effectiveness** | | | | | |
| Log RR of remission in EIP | 0.207 | 0.102 | 0.403 | Normal(𝜇 = 0.207, 𝜎 = 0.470) | Bayesian MA including the cohort data |
| Log RR of relapse in EIP | -0.342 | -0.573 | -0.104 | Normal(𝜇 = -0.342, 𝜎 = 0.143) | Bayesian MA including the cohort data |
| Log RR of remission with clozapine | 0.194 | 0.027 | 0.347 | Normal(𝜇 = 0.198, 𝜎 = 0.238) | ^12^ |
| Log RR of inpatient care in EIP | -0.113 | -0.204 | -0.026 | Normal(𝜇 = -0.113, 𝜎 = 0.055) | ^13^ |
| **Mortality** | | | | | |
| Log SMR of people with psychosis | 1.125 | 1.068 | 1.183 | Normal(𝜇 = 1.125, 𝜎 = 0.035) | ^4^ |
| Log SMR of people with schizophrenia (calibrated) | 1.095 | 1.058 | 1.134 | Normal(𝜇 = 1.095, 𝜎 = 0.023) | Calibration |
| **Health utilities** | | | | | |
| Utility of FEP and relapses | 0.34 | 0.165 | 0.524 | Trunc Normal(b = 1, 𝜇 = 0.34, 𝜎 = 0.109) | ^15^ |
| Utility of remission | 0.80 | 0.745 | 0.853 | Trunc Normal(b = 1, 𝜇 = 0.80, 𝜎 = 0.033) | ^16^ |
| Utility of resistance | 0.65 | 0.222 | 0.935 | Trunc Normal(b = 1, 𝜇 = 0.65, 𝜎 = 0.26) | ^14^ |
| Utility of PNS | 0.69 | 0.553 | 0.821 | Trunc Normal(b = 1, 𝜇 = 0.69, 𝜎 = 0.079) | ^13^ |
| **Costs** | | | | | |
| Per diem inpatient care costs | 30,883 | - | - | fixed | EVC |
| probability of being admitted | 0.357 | 0.109 | 0.661 | beta(𝛼 = 2.50, 𝛽 = 4.45) | Experts elicitation |
| Average length of stay | 42.98 | 30.19 | 56.04 | gamma(*k* = 31.04, 𝜃 = 1.384) | ^17–19^ |
| Costs of relapses CMHT^3^ | $577,315.00 | $334,585.10 | $883,570.60 | gamma(*k* = 11.1, 𝜃 = 51958.35) | EVC |
| Costs of remission CMHT | $184,924.00 | $108,352.20 | $269,499.40 | gamma(*k* = 11.1, 𝜃 = 16643.16) | EVC |
| Costs of resistance CMHT | $820,537.00 | $457,028.40 | $1,257,727.70 | gamma(*k* = 11.1, 𝜃 = 73848.33) | EVC |
| Costs of PNS CMHT | $447,584.00 | $243,981.50 | $691,342.10 | gamma(*k* = 11.1, 𝜃 = 40282.56) | EVC |
| Costs of relapses EIP^3^ | $798,403.00 | $469,608.00 | $1,228,553.00 | gamma(*k* = 11.1, 𝜃 = 71856.27) | EVC |
| Costs of remission EIP | $243,180.00 | $136,939.90 | $372,444.20 | gamma(*k* = 11.1, 𝜃 = 21886.2) | EVC |
| Costs of resistance EIP | $1,045,925.00 | $586,884.90 | $1,595,074.00 | gamma(*k* = 11.1, 𝜃 = 94133.25) | EVC |
| Costs of PNS EIP | $647,775.00 | $362,028.00 | $1,005,818.00 | gamma(*k* = 11.1, 𝜃 = 58299.75) | EVC |
| FEP: first-episode psychosis, PNS: persistent negative symptoms, CMHT: community-mental health teams, EIP: early intervention in psychosis, EVC: estudio de verificacion de costos (unit-costs verification study), RR: risk ratio, SMR: standardised mortality rate, MA: meta-analysis, 𝜇 : mean, 𝜎: standard deviation, k: shape, 𝜃: scale.  1. Parametrisation can differ from chapter 7 because of rescaling to 3-month cycle.  2. Assumed equal to probability of PNS in acute episode.  3. Does not include hospitalisation. Cost of hospitalisation was added as a probabilistic parameter in the model. | | | | | |

# Bayesian synthesis

A principle of evidence-based medicine (EBM) is to use the best available evidence to inform a decision. Initial formulations of the EBM recognised a rigid hierarchy of evidence, relying almost entirely on randomised-controlled trials (RCTs) and systematic reviews of RCTs to make decisions. Recent conceptualisations, however, give higher value to well-conducted observational studies. While RCTs are still the gold standard to estimate causal effects, observational studies are valued for their potential higher generalisability.

When both types of evidence are available, the question of how to combine them arises. Disregarding observational evidence has the downside of excluding important pieces of information, sometimes with greater generalisability. However, including potentially more biased sources of evidence might, to some extent, affects the results of a decision.

Based on the results of previous methodological reviews ^17–19^, we combined the Chilean cohort data with published RCTs using the approach known as power prior. ^20–22^ The list of studies meta-analysed with the cohort data can be found in Table S2.

Briefly, it consists on rising the likelihood of the observational data to a power $\boldsymbol{\alpha}$, which controls the influence of these data on the whole estimate (Eq. 1). A value of $\boldsymbol{\alpha}$ = 0 means downweighing the observational evidence completely. In other words, discarding it. Meanwhile, an $\boldsymbol{\alpha}$ of 1 means the observational evidence is taken as an additional trial.

| $\boldsymbol{P}\left( \boldsymbol{\theta} \vert\boldsymbol{data} \right)\mathcal{\propto L}\left( \boldsymbol{\theta} \vert\boldsymbol{RCT} \right)\mathcal{\times L}\left( \boldsymbol{\theta} \vert\boldsymbol{Obs} \right)^{\boldsymbol{\alpha}}\boldsymbol{\times P(\theta)}$ | (1) |
| --- | --- |

Where $\boldsymbol{P}\left( \boldsymbol{\theta} | \boldsymbol{data} \right)$ is the posterior distribution for the parameter of interest (e.g. log odds of relapse).

$\mathcal{L}$ is the likelihood function for the RCTs and the Observational evidence included in the synthesis

$\boldsymbol{\alpha}$ is a different level of weighting the observational evidence.

In our base case, we conducted our analyses considering the cohort data as an additional trial with an $\alpha$ of 0.5.

Table S2 Studies informing aggregate data

| Study | Reference | Country | Duration (months) | Sample size |
| --- | --- | --- | --- | --- |
| LEO | (Craig et al., 2004) | UK | 18 | 144 |
| OPUS | (Petersen et al., 2005) | Denmark | 24 | 369 |
| OTP | (Grawe et al., 2006) | Norway | 24 | 50 |
| Valencia-12 | ^26^ | Mexico | 12 | 88 |
| PIANO | ^27^ | Italy | 9 | 444 |
| Valencia-6 | ^28^ | Mexico | 6 | 120 |
| JCAP | ^29^ | Japan | 18 | 77 |
| LEO: Lambeth Early Onset, OPUS: Specialized assertive intervention (Danish) , OTP: Optimal Treatment Project, PIANO: Psychosis early Intervention and Assessment of Needs and Outcome, JCAP: Japanese Comprehensive Approach for First-episode Psychosis. | | | | |

# Expert elicitation

## Justification

We followed Iglesias et al.(2016) ^30^ to define “expert elicitation” as the formal process of obtaining quantitative estimations from experts’ judgements about some parameters in the model.

After completing the model conceptualisation phase and while embedding the evidence within the decision model, we found no local evidence for certain parameters in the literature. Furthermore, no information about these parameters was present in the Chilean cohort data.

The parameters mentioned were: a) the proportion of treatment-resistant schizophrenia patients receiving clozapine; b) the incidence of persistent negative FEP; c) the number of people receiving inpatient care during a relapse. Although there was some evidence from the international literature about these parameters, the wide range of estimates might have reduced the applicability of the decision model to the Chilean context. For instance, there is evidence that clozapine prescription varies widely between countries, with the prevalence of clozapine use as high as 189.2/100,000 persons in Finland to 0.6/100,000 persons in Japan ^31^. Similarly, rates of hospitalisation and length-of-stay are highly dependent on local practices and guidelines ^32^.

Hence, obtaining an estimate that reflected these uncertainties from Latin American experts was considered necessary for the model development.

## Methods

The elicitation exercise followed the Reporting guidelines for Expert Judgment ^30^ and the recommendations of the Guidance on Expert Knowledge Elicitation published by the European Food Safety Authority (EFSA).^34–36^

### Experts selection

For the present elicitation task, experts were invited to participate according to the following criteria: (a) participants were all familiar with EIP services in Latin America as a result of their clinical and/or research practice; (b) they all had a background in conducting research in the field of EIP; (c) experts should collectively cover knowledge regarding various settings from Latin America, including different countries and levels of care, given the impact of implementing specialists services on upstream and downstream health services.

### Evidence dossier

An evidence dossier was circulated by email one month before the elicitation exercise. The dossier included a description of the model, definitions and concepts relevant to the parameters to be elicited and international evidence about such quantities. The dossier also included an explanation of the elicitation exercise.

A reminder of the exercise was sent one week before the event.

### Elicitation exercise

This Delphi panel took place in the first meeting of the Latin American network for the study of early psychosis. This network aims to develop regionally-relevant knowledge in the early stages of psychotic disorders, including approaches from biological to clinical and social sciences ^41^. It includes researchers from six Latin American countries (Argentina, Bolivia, Brazil, Chile, Colombia and Mexico).

During the meeting, a slide presentation was given to remind the research question and evidence about parameters. After that, instructions of the exercise were given with time to discuss and resolve questions. This session was facilitated by two authors of the paper (DA and EU). The parameters were framed in natural numbers to improve responses of those with less probabilistic thought. For instance, instead of asking about quantiles and likelihood, the experts were asked to say how many patients out of 20 would receive clozapine if they developed treatment-resistant schizophrenia. They were asked to specify their best estimate, as well as their lower and upper bounds estimate. Experts were told to gauge their boundaries assuming the chances of observing numbers outside those ranges would be very unlikely. The answers were then presented as proportions and again contrasted with the experts to check if they truly represented their beliefs.

Questions were given in paper format translated into Spanish, Portuguese and English. During the first round, the participants were asked to make questions and comments, but without sharing their estimations with their colleagues. The second round took place the next day. The first-round estimates and histograms were presented and again participants were allowed to make questions and comments. After a brief discussion, experts gave their estimates again.

### Statistical analysis

The statistical analysis used the upper and lower estimates elicited from experts as the first and third quantiles, respectively. The “most likely value” was considered as the median and since the elicited parameters were probabilities, a natural bound between 0 and 1 was used. Remaining quantiles were fitted based on a simple normal approximation by setting $E(X)$ equal to the 0.5th quantile, and the $Var(X) = \left( 0.6 quantile - 0.4 quantile \right)^{2} / 0.25.$ Intermediate quantiles and fitted distribution were obtained using the SHELF R package. This package has been specifically designed to fit parametric distributions using least squares on the cumulative distribution function.^42^

Finally, in order to minimise the effect of overconfident responders and to include the wide range of practices, we decided to pool multiple answers by applying equal weight to each expert probability distribution.

### Ethical issues

No identifiable data was obtained from the experts. Therefore, no informed consent was considered necessary. Nonetheless, the participation was entirely voluntary in both rounds. The project also received King’s College London ethical approval.

## Results

### The exercise

The first round included 31 people (32.2% female) with a median (range) age of 38 (28 – 63) years and a median (range) of 12 (4 – 40) years of experience. Twenty-three of them worked mostly as clinicians and 8 worked mainly in research. A 69.5% of participants reported working in both areas. 23 experts participated in the second round, with a higher representation of men (78.3%) and clinicians (78.3%). All the Latin American countries which are members of the network were represented in both rounds. Namely, Argentina, Brazil, Bolivia, Chile, Colombia and Mexico.

Table S3. Characteristics of participant experts

|  | **First round** | **Second Round** |
| --- | --- | --- |
| Sample size (N) | 31 | 23 |
| Age (years) |  |  |
| Mean (SD) | 40 (8.44) | 40.6 (8.67) |
| Experience (years) |  |  |
| Mean (SD) | 14.6 (9.31) | 14.2 (8.29) |
| Gender |  |  |
| Female N (%) | 10 (32.3%) | 5 (21.7%) |
| Male N (%) | 21 (67.7%) | 18 (78.3%) |
| Main activity |  |  |
| Clinician | 23 (74.2%) | 18 (78.3%) |
| Non-clinical researcher | 8 (25.8%) | 5 (21.7%) |

### Data aggregation

Figure S2 shows the experts’ best estimates during the first round. The first and second question had the higher dispersion of estimates, with a median (range) of 12 (1 – 20) out of 20 patients receiving clozapine and 6 (2 – 20) out of 20 patients developing persistent negative symptoms after FEP, respectively. The third question (hospitalisation after a relapse) resulted in a higher agreement, with a median (range) of 5 (2 – 10) out of 20 patients receiving inpatient care according to the experts.

Figure S2. Distribution of responses after the first round

| 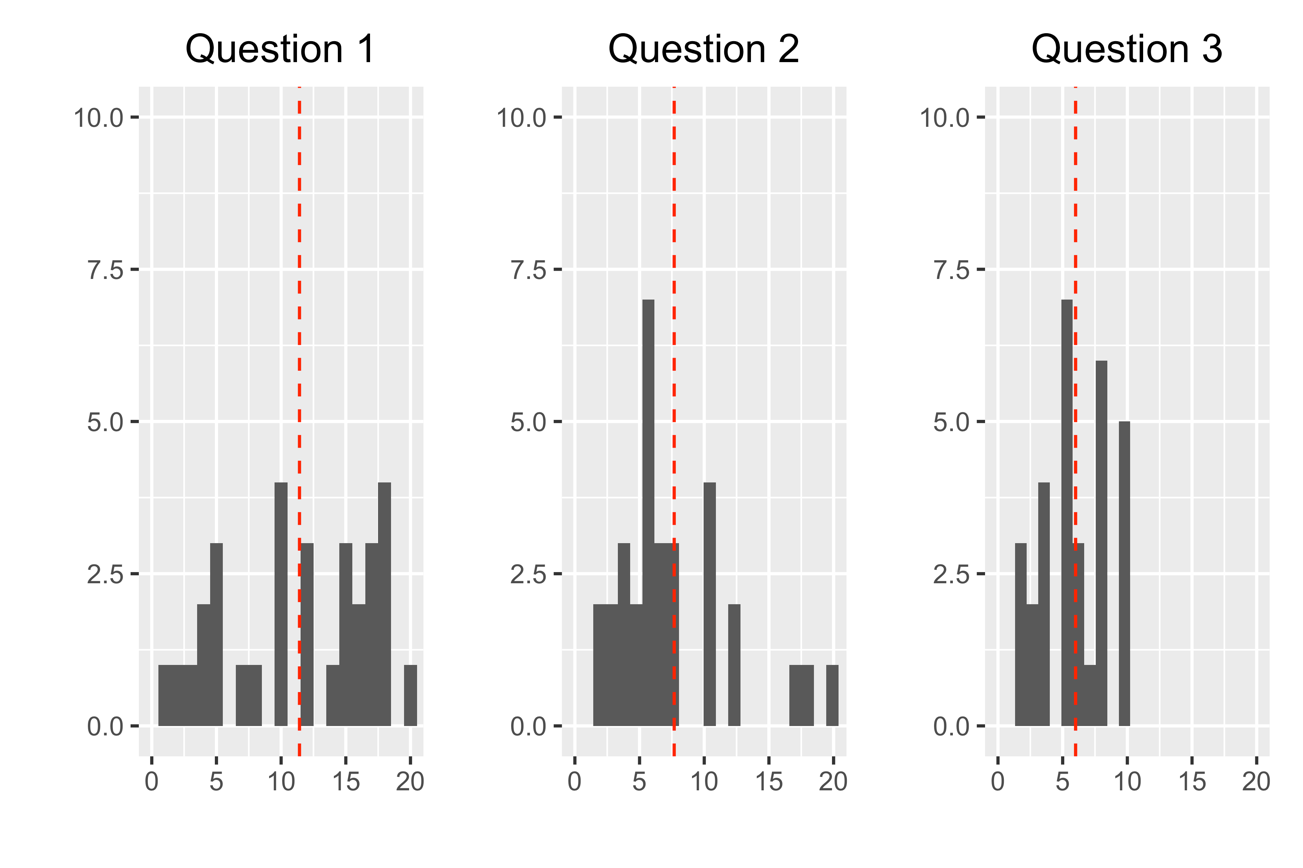 |
| --- |
| **Histograms showing the distribution of experts’ responses. The x-axis represents the number of experts, while the y-axis shows the range of plausible answers between 0 and 20. Red dashed-lines represent the mean pooled estimate.**  **Question 1: clozapine use in patients with treatment-resistant schizophrenia.**  **Question 2: number of people with persistent negative symptoms after a first-episode psychosis.**  **Question 3: number of patients receiving inpatient care after a relapse.** |

The distribution of experts’ best estimate from the second round is shown in Figure S3. Compared to the responses in the first round, there was higher agreement among the experts in questions 2 and 3. The median (range) of patients developing persistent negative symptoms after a FEP was 6 (4 – 8) out of 20 according to the experts, while the median (range) of 6 (5 – 8) out of 20 patients are hospitalised after a relapse according to the experts’ judgements. The responses to question 1 remained relatively spread throughout the range given to the experts, with a median (range) of 12 (10 – 18) out of 20 treatment-resistant schizophrenia patients receiving clozapine as judged by the participants.

Figure S3. Distribution of responses after the second round

| 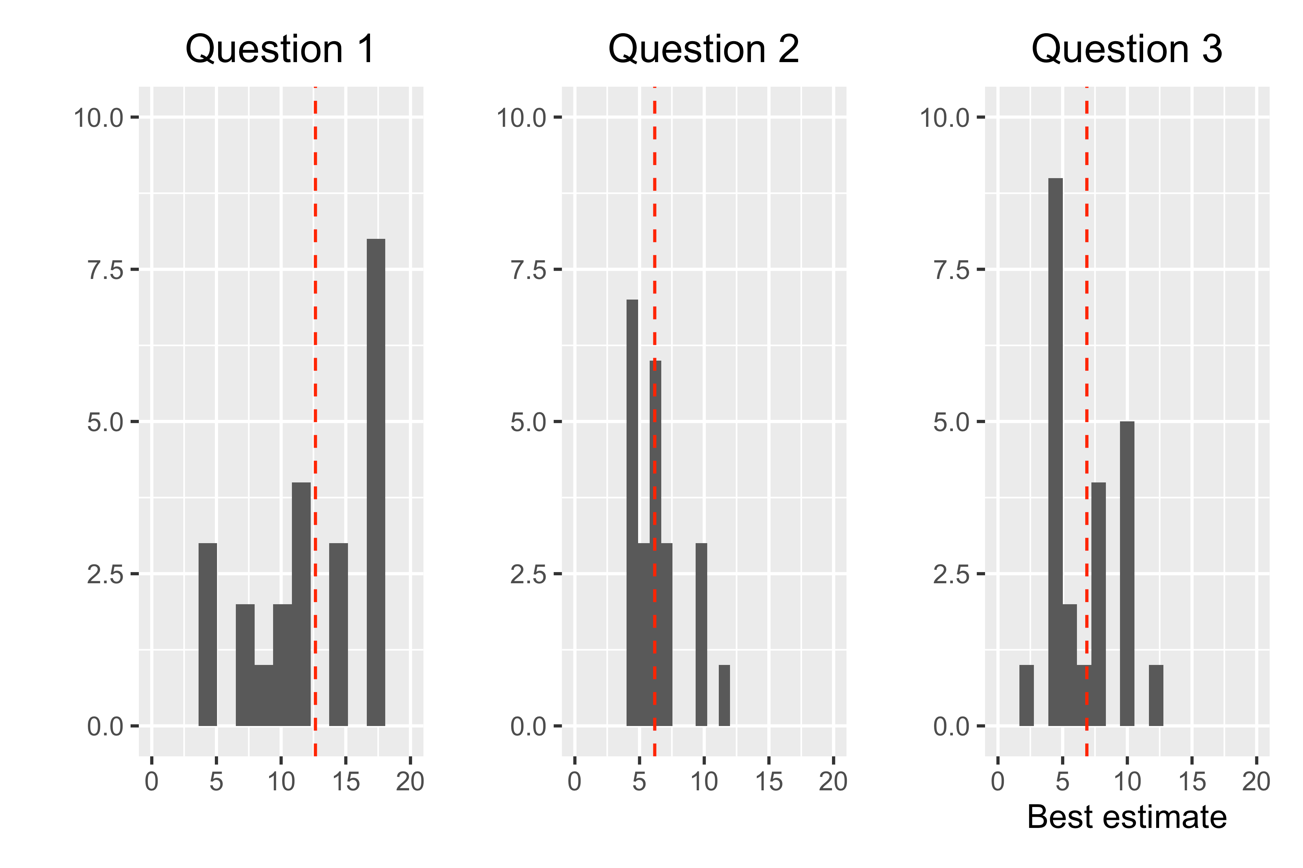 |
| --- |
| **Histograms showing the distribution of experts’ responses. The x-axis represents the number of experts, while the y-axis shows the range of plausible answers between 0 and 20. Red dashed-lines represent the mean pooled estimate.**  **Question 1: clozapine use in patients with treatment-resistant schizophrenia.**  **Question 2: number of people with persistent negative symptoms after a first-episode psychosis.**  **Question 3: number of patients receiving inpatient care after a relapse.** |

Figure S4 shows the best estimates, as well as lower and upper estimates given by individual experts in the second round of the exercise. The figure depicts differences among participants between answers, but also the degree of uncertainty (or confidence) within the same question.

Figure S4. Distribution of individual experts' judgments on each question

| 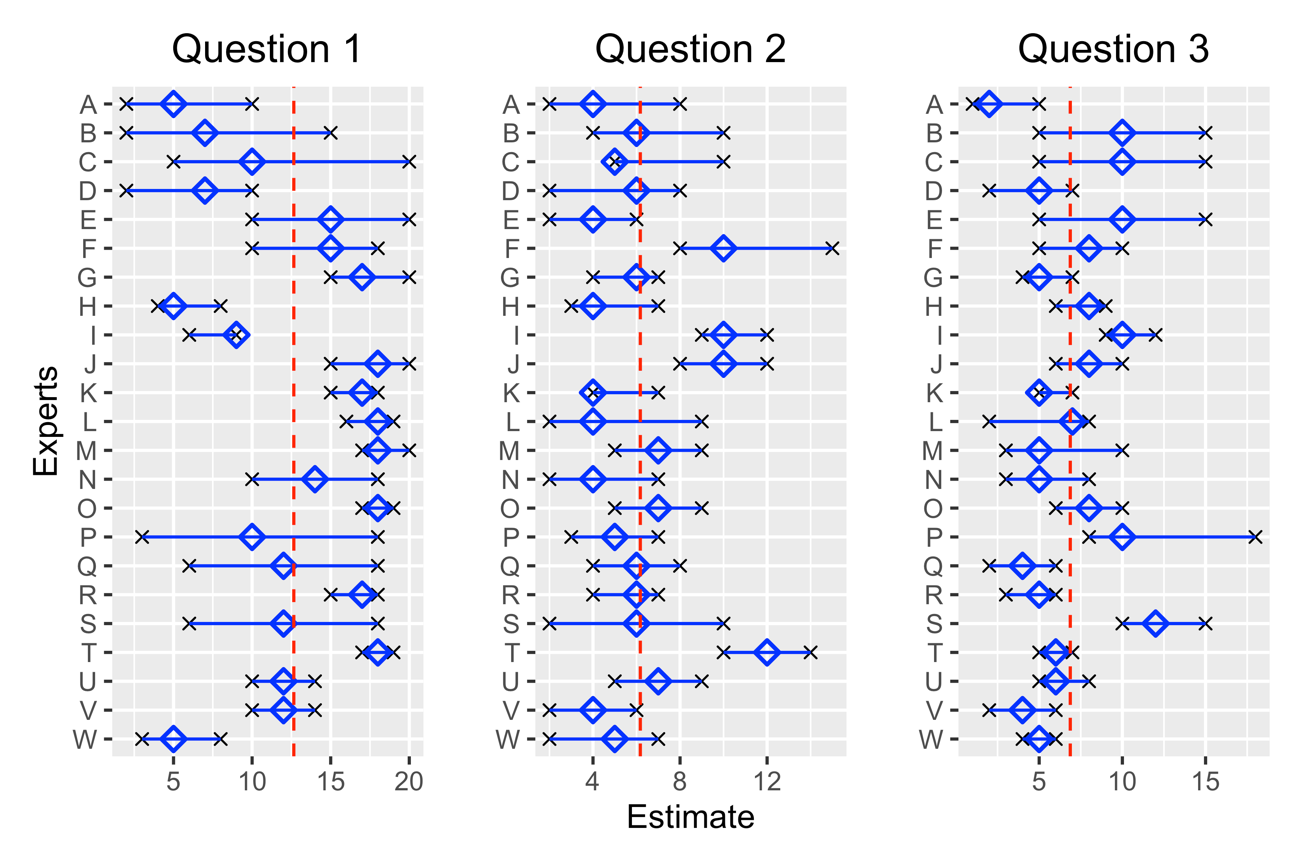 |
| --- |
| **Distribution of responses by experts. The x-axis shows each individual experts, while the y-axis shows the range of plausible answers between 0 and 20. Diamonds represent the experts’ best estimates, while “x” represent their plausible ranges. Red dashed-lines represent the mean pooled estimate.**  **Question 1: clozapine use in patients with treatment-resistant schizophrenia.**  **Question 2: number of people with persistent negative symptoms after a first-episode psychosis.**  **Question 3: number of patients receiving inpatient care after a relapse.** |

The final distribution obtained from pooling the best estimate using equal weighting from the experts can be found in Figure S5. Parameters for questions 2 and 3 were best fitted using beta distributions according to the sum of squares criterion. The distribution fitted to parameter 2 and 3 is α=2.49, β=5.04 and α=2.5, β=4.45 respectively. In the case of question 1, a Student-t (0.653, 022) with 3 degrees of freedom best fitted the data using the sum of squares criterion. However, applying this distribution to the decision model would have resulted in sampling values outside of the plausible range. Therefore, the second best option that minimised the sum of squares was used instead. This resulted in a Beta distribution (α=2.37, β=1.45).

Figure S5. Fitted distributions obtained from pooling experts' opinions.


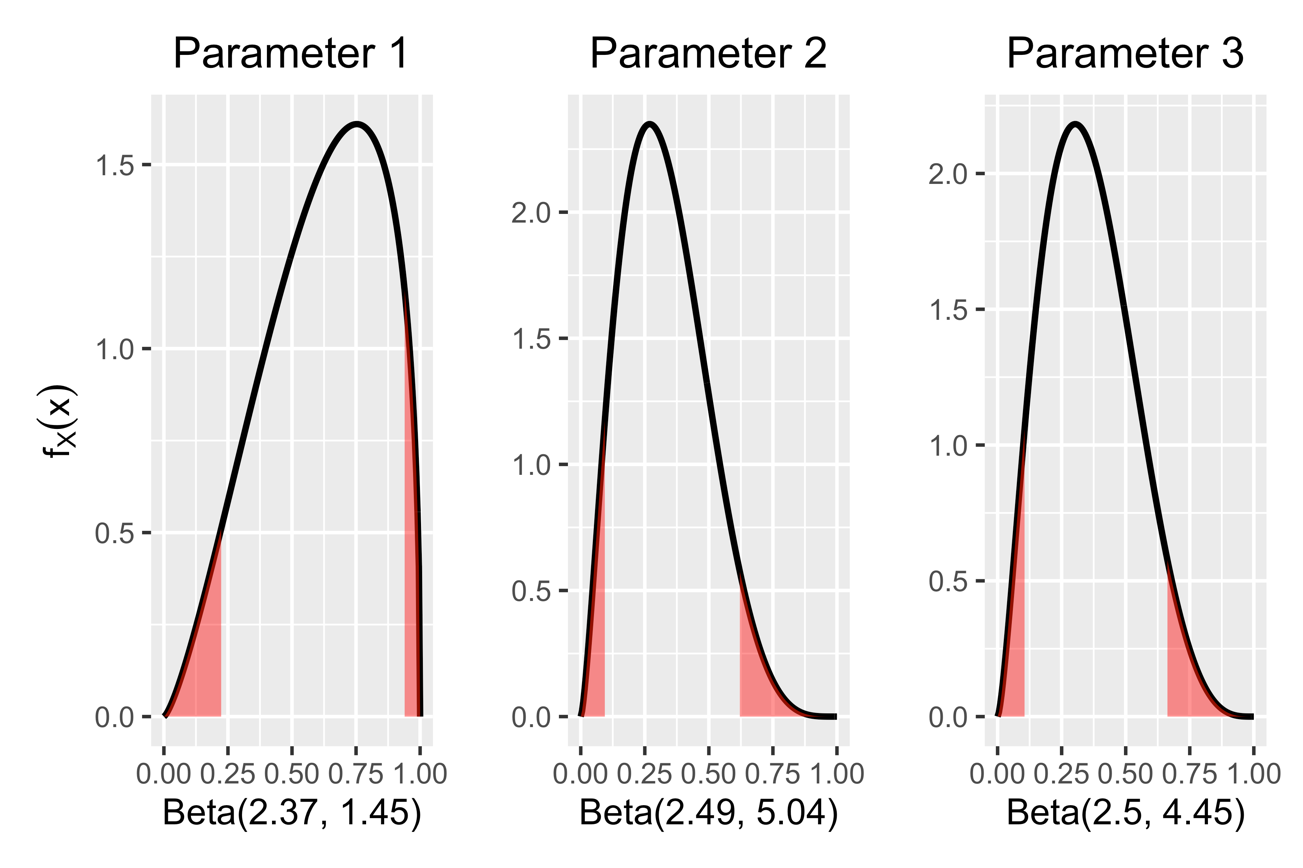


### Usage of elicited quantities in the model

The mean values of each elicited distribution were used in the base case analysis of the decision model. The whole distributions, reflecting the uncertainty expressed by the experts, were embedded in the probabilistic sensitivity analysis.

As the prevalence of persistent negative symptoms in people with FEP was elicited using a one-year timeframe, we rescaled the parameter to a 3-month period assuming constant rate throughout the year. ^43,44^

# List of black-box and white-box tests

Table S4 List of model tests conducted

| Test description | Result |
| --- | --- |
| Pre-analysis calculations | |
| The probability of an event increases when a higher RR is used? | Yes |
| Are the treatment effect inputs within plausible ranges? | Yes |
| States calculations | |
| Sum the proportion of patients in all health states at any model time point | Total proportions equated 1.0 |
| Applying a lifetime horizon and checking the final state of the cohort | All patients were dead at the end of the simulation |
| Set all costs to 0 | Total costs equal to zero. |
| Set all utilities to 0 | Total QALYs equal to zero. |
| Set all utilities to 1 | QALYs gained were the same as the LYGs |
| Result calculations | |
| Set QALY discount rate to 0 | The discounted and undiscounted QALY gained were the same |
| Set cost discount rate to 0 | The discounted and undiscounted costs were the same |
| Changes in LYGs, QALYs and costs with different time horizons | Lower LYGs, QALYs and costs for shorter time horizons |
| Uncertainty analysis calculations | |
| All parameters used in the sensitivity analysis have an appropriate associated distribution | Yes |
| PSA cloud with unusual shape? | No |
| RR: risk ratio, QALY: quality-adjusted life year, LYG: life-years gained, PSA: probabilistic sensitivity analysis, ICER: incremental cost-effectiveness ratio | |

# Deterministic sensitivity analysis

The results of the deterministic sensitivity analysis are presented in Figure S6. The tornado plot ranks the parameters with higher impact on the net monetary benefit. The model was sensitive to the following parameters: the probability of receiving inpatient care, the utility values of remission, the utility values of relapses, the effectiveness of the intervention in reducing relapses and the costs associated with relapses and remission. Despite these changes. The incremental NMB favoured EIP, even at the lowest willingness to pay of 0.5 GDPpc. As a result, EIP remained as the most cost-effective alternative compared to CMHTs.

| Figure S6 Deterministic sensitivity analysis on the net monetary benefit  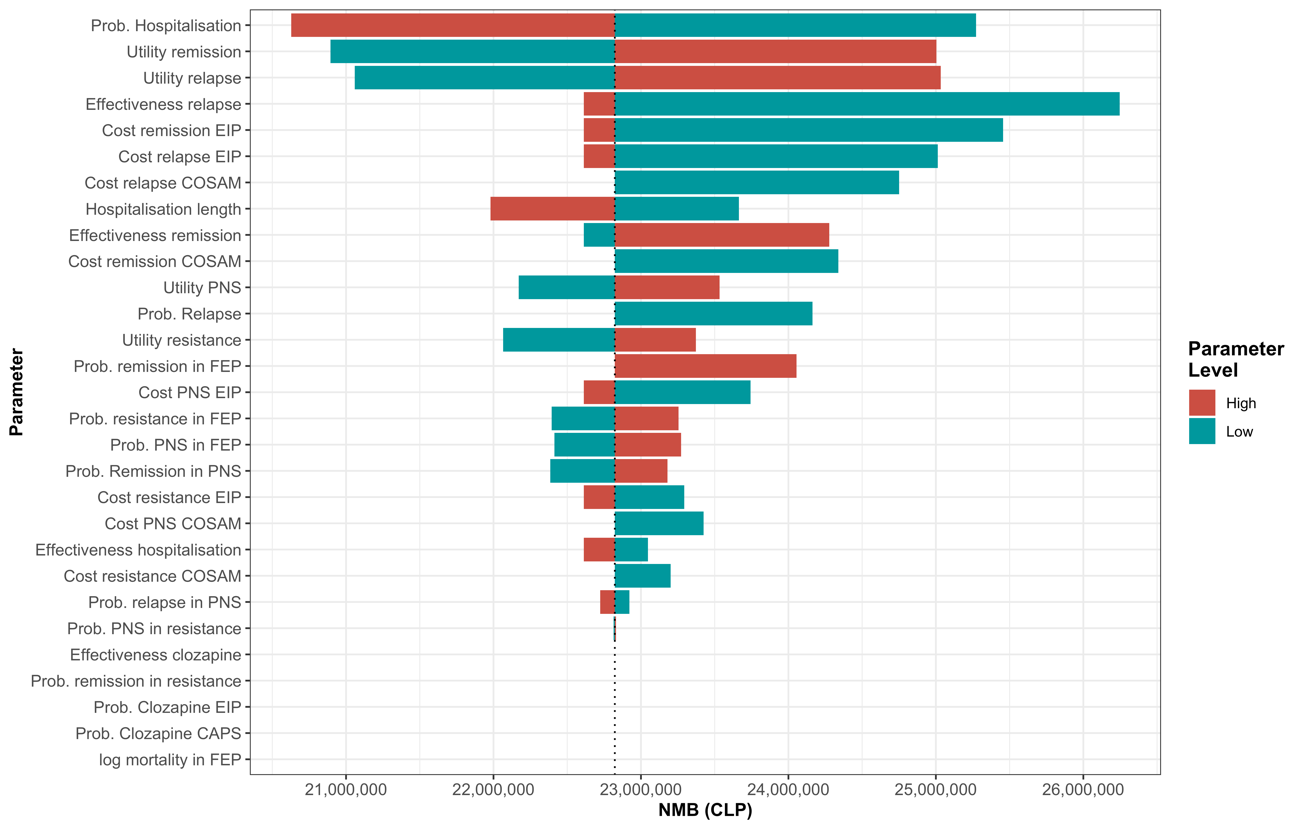 |
| --- |
| **List of parameters tested in the one-way deterministic sensitivity analysis. Each bar represent the change in the NMB estimated with different values of each parameter. Red colour represents higher values of the parameter, while green colour represents low values. On each sensitivity analysis the 95% credible interval was tested.**  **Prob: probability, EIP: Early Intervention in Psychosis, CMHTS: Community mental health teams, FEP: First-episode psychosis, PNS: persistent negative symptoms, NMB: net monetary benefit, CLP: Chilean peso.** |

# Value of information analysis

The overall EVPI per person affected by the decision was estimated at CLP 805,857.42 (£1,374.44). Assuming an annual incidence of FEP of 3240 patients ^45^, and a decision relevance time horizon of 10 years, the overall expected value of removing decision uncertainty would be CLP 26.1 thousand million (£44,532,358.04) in total.

| Figure S7 Population expected value of perfect information curve  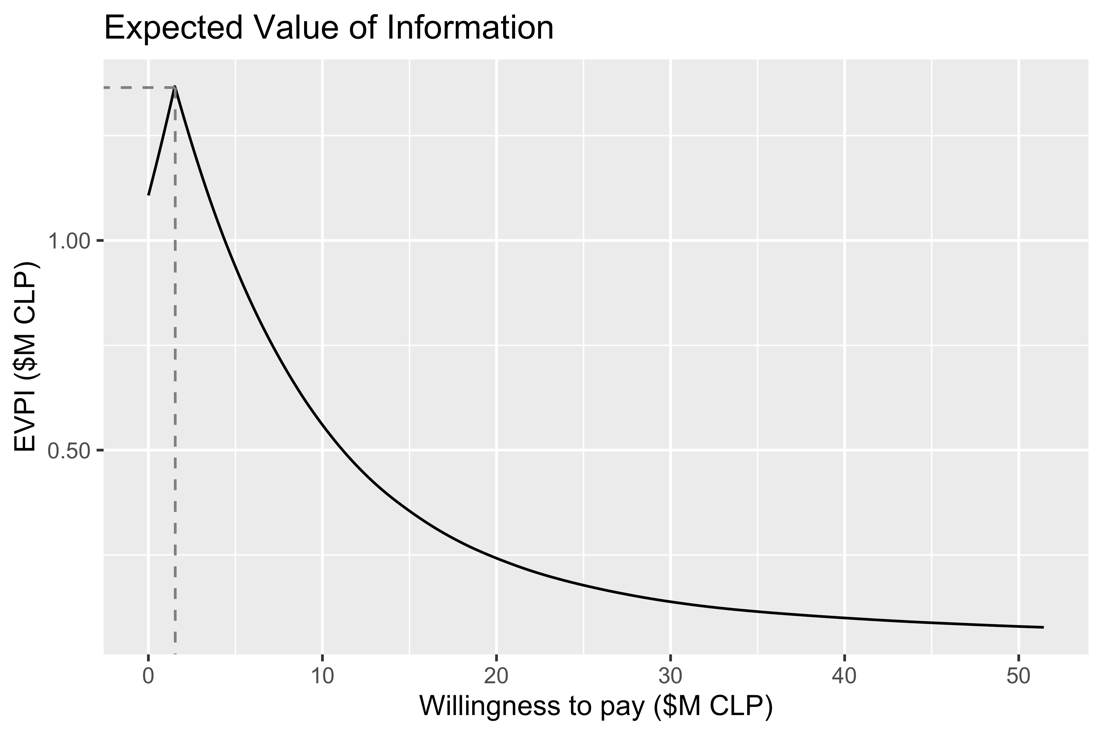 |
| --- |
| **Relationship between the EVPI in million CLP and the WTP (also in million CLP). The value of new information increases up to the cost-effectiveness threshold where the decision uncertainty is highest. Higher values of WTP reduce the EVPI because the decision is more certain and therefore new evidence is considered less valuable.**  **EVPI: expected value of perfect information; CLP: Chilean pesos; WTP: willingness to pay.** |

Additionally, the expected value of partial perfect information (EVPPI) analysis suggested that the parameters causing most of the decision uncertainty were the effectiveness of the intervention in reducing relapses, the costs of managing those relapses, the effectiveness of the intervention promoting remission and the costs associated with the remission state.

Finally, Figure S8 shows the expected value of sampling information and the expected net benefit of sampling an additional study with different sample sizes for the parameters causing most of the uncertainty in the model. The figure also shows the optimal sample size (OSS) to maximise new information. For instance, if the additional study was observational, the OSS would be 500 participants. Conversely, if the study to be conducted was a RCT, the OSS required would be 280 participants.

| Figure S8 Expected net benefit of additional research  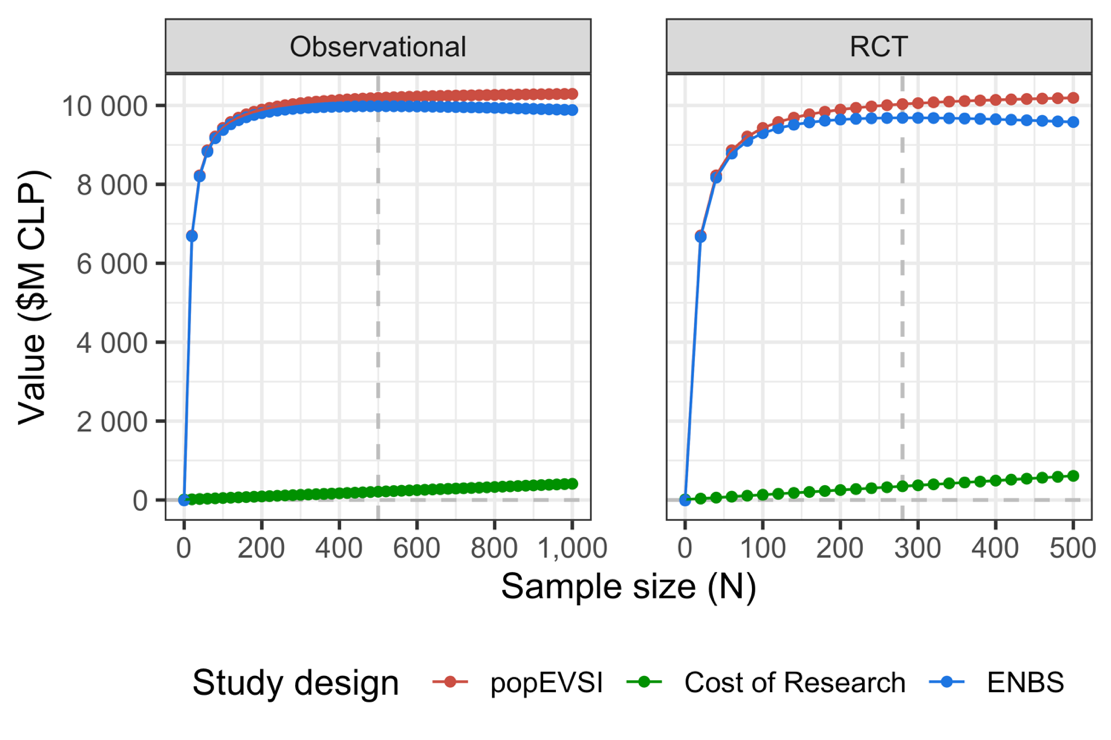 |
| --- |
| **This figure shows the costs (in $M CLP) of conducting a new study, either observational (left) or a RCT (right) and their respective sample sizes. The green lines show a linear increment in costs as the sample size increases. The red lines represent the population EVSI and the blue lines the ENBS. The dotted vertical line represents the point at which the ENBS is maximum and the sample size is optimum.**  **CLP: Chilean peso; EVSI: expected value of sampling information; expected net benefit of sampling; RCT: randomised controlled trial.** |

# Other scenarios

Assuming the intervention has a continuous effect increased the chances of EIP being the most cost-effective alternative. Under this scenario, the incremental costs of EIP services decreased to CLP 399,654, while the incremental QALYs gained rose to 0.33. As a result, the resulting ICER was reduced to CLP 1,187,422 per QALY, compared to the base case ICER of CLP 5,550,044 per QALY.

Assuming longer time-horizons had a slightly positive impact on the cost-effectiveness of EIP services. The estimated ICER decreased to CLP 4,817,343 per QALY, CLP 4,577,691 per QALY and 4,290,232 per QALY at 20, 30 and 80 years (lifetime in Chile), respectively.

Finally, including the emerging evidence of the effect of EIP services on mortality ^46^ had practically no effect in the base case analysis (ICER: CLP 5,335,612 per QALY). However, using a lifetime time horizon reduced the ICER to CLP 3,255,353 per QALY.

# References

1 Jin H, Tappenden P, MacCabe JH, Robinson S, Byford S. Evaluation of the Cost-effectiveness of Services for Schizophrenia in the UK Across the Entire Care Pathway in a Single Whole-Disease Model. *JAMA Netw Open* 2020; **3**: e205888.

2 Fusar-Poli P, McGorry PD, Kane JM. Improving outcomes of first-episode psychosis: an overview. *World Psychiatry* 2017; **16**: 251–65.

3 McGorry PD, Killackey E, Yung A. Early intervention in psychosis: concepts, evidence and future directions. *World Psychiatry* 2008; **7**: 148–56.

4 Oakley P, Kisely S, Baxter A, Harris M, Desoe J, Dziouba A, *et al.* Increased mortality among people with schizophrenia and other non-affective psychotic disorders in the community: A systematic review and meta-analysis. *J Psychiatr Res* 2018; **102**: 245–53.

5 The Royal College of Psychiatrists. National Clinical Audit of Psychosis – National Report for the Core Audit 2018. Healthcare Quality Improvement Partnership., 2018 (https://www.rcpsych.ac.uk/docs/default-source/improving-care/ccqi/national-clinical-audits/ncap-library/ncap-national-report-for-core-audit-2018.pdf?sfvrsn=23c6a262_2).

6 Correll CU, Galling B, Pawar A, Krivko A, Bonetto C, Ruggeri M, *et al.* Comparison of Early Intervention Services vs Treatment as Usual for Early-Phase Psychosis: A Systematic Review, Meta-analysis, and Meta-regression. *JAMA Psychiatry* 2018; **75**: 555–65.

7 Mena C, Gonzalez-Valderrama A, Iruretagoyena B, Undurraga J, Crossley NA. Early treatment resistance in a Latin-American cohort of patients with schizophrenia. *Schizophr Res* 2018; **199**: 380–5.

8 Alvarez-Jiménez M, Parker AG, Hetrick SE, McGorry PD, Gleeson JF. Preventing the second episode: a systematic review and meta-analysis of psychosocial and pharmacological trials in first-episode psychosis. *Schizophr Bull* 2011; **37**: 619–30.

9 Siskind D, Siskind V, Kisely S. Clozapine Response Rates among People with Treatment-Resistant Schizophrenia: Data from a Systematic Review and Meta-Analysis. *Can J Psychiatry* 2017; **62**: 772–7.

10 Buchanan RW, Panagides J, Zhao J, Phiri P, den Hollander W, Ha X, *et al.* Asenapine versus olanzapine in people with persistent negative symptoms of schizophrenia. *J Clin Psychopharmacol* 2012; **32**: 36–45.

11 Doyle R, Behan C, OʼKeeffe D, Masterson S, Kinsella A, Kelly A, *et al.* Clozapine Use in a Cohort of First-Episode Psychosis. *J Clin Psychopharmacol* 2017; **37**: 512–7.

12 Siskind D, McCartney L, Goldschlager R, Kisely S. Clozapine v. first- and second-generation antipsychotics in treatment-refractory schizophrenia: systematic review and meta-analysis. *Br J Psychiatry* 2016; **209**: 385–92.

13 Randall JR, Vokey S, Loewen H, Martens PJ, Brownell M, Katz A, *et al.* A Systematic Review of the Effect of Early Interventions for Psychosis on the Usage of Inpatient Services. *Schizophr Bull* 2015; **41**: 1379–86.

14 Aceituno D, Pennington M, Iruretagoyena B, Prina AM, McCrone P. Health State Utility Values in Schizophrenia: A Systematic Review and Meta-Analysis. *Value Health* 2020; **23**: 1256–67.

15 Garcia-Gordillo MA, Collado-Mateo D, Olivares PR, Adsuar JC. Chilean population norms derived from the health-related quality of Life SF-6D. *Eur J Health Econ* 2018; **19**: 675–86.

16 Davies LM, Barnes TRE, Jones PB, Lewis S, Gaughran F, Hayhurst K, *et al.* A randomized controlled trial of the cost-utility of second-generation antipsychotics in people with psychosis and eligible for clozapine. *Value Health* 2008; **11**: 549–62.

17 Ades AE, Welton NJ, Caldwell D, Price M, Goubar A, Lu G. Multiparameter evidence synthesis in epidemiology and medical decision-making. *J Health Serv Res Policy* 2008; **13 Suppl 3**: 12–22.

18 Riley RD, Steyerberg EW. Meta-analysis of a binary outcome using individual participant data and aggregate data. *Res Synth Methods* 2010; **1**: 2–19.

19 Verde PE, Ohmann C. Combining randomized and non-randomized evidence in clinical research: a review of methods and applications. *Res Synth Methods* 2015; **6**: 45–62.

20 Ibrahim JG, Chen M-H, Gwon Y, Chen F. The power prior: theory and applications. *Stat Med* 2015; **34**: 3724–49.

21 Spiegelhalter DJ, Abrams KR, Myles JP. *Bayesian Approaches to Clinical Trials and Health-Care Evaluation*. John Wiley & Sons, 2004.

22 Welton NJ, Sutton AJ, Cooper N, Abrams KR, Ades AE. *Evidence Synthesis for Decision Making in Healthcare*. John Wiley & Sons, 2012.

23 Craig TKJ, Garety P, Power P, Rahaman N, Colbert S, Fornells-Ambrojo M, *et al.* The Lambeth Early Onset (LEO) Team: randomised controlled trial of the effectiveness of specialised care for early psychosis. *BMJ* 2004; **329**: 1067.

24 Petersen L, Jeppesen P, Thorup A, Abel M-B, Øhlenschlaeger J, Christensen TØ, *et al.* A randomised multicentre trial of integrated versus standard treatment for patients with a first episode of psychotic illness. *BMJ* 2005; **331**: 602.

25 Grawe RW, Falloon IRH, Widen JH, Skogvoll E. Two years of continued early treatment for recent-onset schizophrenia: a randomised controlled study. *Acta Psychiatr Scand* 2006; **114**: 328–36.

26 Valencia M, Juarez F, Ortega H. Integrated treatment to achieve functional recovery for first-episode psychosis. *Schizophr Res Treatment* 2012; **2012**: 962371.

27 Ruggeri M, Bonetto C, Lasalvia A, Fioritti A, de Girolamo G, Santonastaso P, *et al.* Feasibility and Effectiveness of a Multi-Element Psychosocial Intervention for First-Episode Psychosis: Results From the Cluster-Randomized Controlled GET UP PIANO Trial in a Catchment Area of 10 Million Inhabitants. *Schizophr Bull* 2015; **41**: 1192–203.

28 Valencia M, Juarez F, Delgado M, Díaz A, Others. Early intervention to improve clinical and functional outcome in patients with first episode-psychosis. 2017. (https://www.iconceptpress.com/book/mental-disorder/11000123/1305000979.pdf).

29 Nishida A, Ando S, Yamasaki S, Koike S, Ichihashi K, Miyakoshi Y, *et al.* A randomized controlled trial of comprehensive early intervention care in patients with first-episode psychosis in Japan: 1.5-year outcomes from the J-CAP study. *J Psychiatr Res* 2018; **102**: 136–41.

30 Iglesias CP, Thompson A, Rogowski WH, Payne K. Reporting Guidelines for the Use of Expert Judgement in Model-Based Economic Evaluations. *Pharmacoeconomics* 2016; **34**: 1161–72.

31 Bachmann CJ, Aagaard L, Bernardo M, Brandt L, Cartabia M, Clavenna A, *et al.* International trends in clozapine use: a study in 17 countries. *Acta Psychiatr Scand* 2017; **136**: 37–51.

32 Pennington M, McCrone P. The Cost of Relapse in Schizophrenia. *Pharmacoeconomics* 2017; **35**: 921–36.

33 European Food Safety Authority. Guidance on Expert Knowledge Elicitation in Food and Feed Safety Risk Assessment. , 2014.

34 Tversky A, Kahneman D. Judgment under Uncertainty: Heuristics and Biases. *Science* 1974; **185**: 1124–31.

35 O’Hagan A, Buck CE, Daneshkhah A, Richard Eiser J, Garthwaite PH, Jenkinson DJ, *et al.* *Uncertain Judgements: Eliciting Experts’ Probabilities*. John Wiley & Sons, 2006.

36 Dias LC, Morton A, Quigley J. *Elicitation: The Science and Art of Structuring Judgement*. Springer International Publishing, 2018.

37 Cooke RM. Experts in uncertainty: Opinion and subjective probability in science. *Environmental ethics and science policy series* 1991; **321**. (https://psycnet.apa.org/fulltext/1991-98990-000.pdf).

38 Gosling JP. SHELF: The Sheffield Elicitation Framework. In *Elicitation: The Science and Art of Structuring Judgement* (eds LC Dias, A Morton, J Quigley ): 61–93. Springer International Publishing, 2018.

39 Rowe G, Wright G. The Delphi technique as a forecasting tool: issues and analysis. *Int J Forecast* 1999; **15**: 353–75.

40 Grigore B, Peters J, Hyde C, Stein K. Methods to elicit probability distributions from experts: a systematic review of reported practice in health technology assessment. *Pharmacoeconomics* 2013; **31**: 991–1003.

41 Crossley N, Guinjoan S, Rivera G, Jackowski A, Gadelha A, Elkis H, *et al.* S81. ANDES NETWORK – STUDYING EARLY PSYCHOSIS IN LATIN AMERICA. *Schizophr Bull* 2019; **45**: S338–S338.

42 Oakley J. SHELF: Tools to Support the Sheffield Elicitation Framework. 2020. (https://CRAN.R-project.org/package=SHELF).

43 Briggs AH, Claxton K, Sculpher MJ. *Decision modelling for health economic evaluation*. Oxford University Press, 2006.

44 Fleurence RL, Hollenbeak CS. Rates and Probabilities in Economic Modelling. *Pharmacoeconomics* 2007; **25**: 3–6.

45 González-Valderrama A, Jongsma HE, Mena C, Castañeda CP, Nachar R, Undurraga J, *et al.* The incidence of non-affective psychotic disorders in Chile between 2005 and 2018: results from a national register of over 30 000 cases. *Psychol Med* 2020; : 1–10.

46 Chan SKW, Chan HYV, Devlin J, Bastiampillai T, Mohan T, Hui CLM, *et al.* A systematic review of long-term outcomes of patients with psychosis who received early intervention services. *Int Rev Psychiatry* 2019; **31**: 425–40.
